# The Clinical Significance of Delirium in the Intensive Care Unit.

**Published:** 2012-01-18

**Authors:** Claudia Crimi, Luca M. Bigatello

**Affiliations:** Dipartimento di Medicina Interna e Medicina, Specialistica, Sezione di Malattie Respiratorie, Universita’ di Catania, Ospedale Policlinico G. Rodolico, Via S. Sofia 78, 95123 Catania, Italy; Tufts University School of Medicine. Department of Anesthesiology and Pain Medicine, St. Elizabeth’s Medical Center, 736 Cambridge Street, Boston, Massachusetts 02135

**Keywords:** ICU delirium, acute brain dysfunction, diagnosis of ICU delirium

## Abstract

Delirium is prevalent among intensive care unit patients. It prolongs length of stay, increases costs, and is independently associated with higher mortality rates. While its specific biological pathways are largely unknown, environmental and iatrogenic determinants have been repeatedly recognized. Removal of the known triggers and pharmacologic intervention constitute available therapies. This review focuses on the clinical significance of delirium in critically ill patients, from its prevalence to its long-term impact, the ways that we have to diagnose it, and the available therapeutic options.

## Case report

Mr. B. O’ D. is a 63 year old patient who was admitted to the Veterans Administration Boston surgical intensive care unit (ICU) in May 2011 following a hepatic resection for metastatic colon carcinoma. His comorbidity included hypertension, hyperlipidemia and post-traumatic stress disorder (PTSD). His habitual medications consisted of anti-hypertensives, a statin, benzodiazepines, a serotonin-reuptake inhibitor, and a phenothiazine antipsychotic. His six-hour intraoperative course was complicated by a blood loss estimated at 2–3 liters, associated with several episodes of hypotension. He was admitted to the ICU late in the day, planning to extubate his trachea in the morning when fully stabilized. However, every attempt to wean sedative medications resulted in uncontrollable agitation: he was severely *delirious*. A number of pharmacologic interventions were unsuccessfully attempted, including intravenous haloperidol and a continuous infusion of dexmedetomidine. It was subsequently learned that the patient had been consuming for some time substantial doses of unprescribed benzodiazepines. Subsequently, the combination of higher doses of lorazepam and an oral anti-psychotic allowed discontinuing continuous intravenous sedation. After two failed tracheal extubations due to a combination of respiratory failure and delirium, a tracheostomy was performed. The patient was eventually transferred to a rehabilitation facility after 19 days of ICU and 5 additional days on the general ward, with a tracheostomy tube in place. Mr. O’ D.’s case raises a number of relevant issues, including:

1. Delirium prolonged his ICU course, and resulted in the need for a tracheostomy. Overall, his hospital course was much longer than expected, and costs for his care were much higher than would have been otherwise for him, his family and society.
2. Despite the violence of his delirium, he did not injure himself.
3. The least recommended class of drugs for treating delirium, benzodiazepines, was instrumental for his clinical turn-around, although it did not resolve delirium *per se*.
4. The newest and most expensive drug, dexmedetomidine, was inconsequential.
5. A number of factors may have contributed to the onset of delirium, including hypotension, tracheal intubation, and benzodiazepine withdrawal. Although not reported in the literature to our knowledge, one wonders about the role of PTSD as a promoter of delirium.

## Introduction

Delirium can be a frightening experience for patients and their families. In its most obvious manifestations of psychomotor agitation it can directly harm the patient and occasionally the caregivers. (1) Its fluctuating character is such that patients have period of lucidity between attacks, and may vividly remember the nightmares and delusions that they experienced. This striking feature is a likely contributor to the development of ‘post-ICU stress disorder’ that may haunt patients for years after the resolution of critical illness. (2) Over the past decade, we have learned that delirium in ICU patients is associated with an increased morbidity, prolonged length of stay, long-term psycho-social harm, and possibly a higher mortality rate. (3,4) This review focuses on the significance of delirium in critically ill patients, from its prevalence to its long-term impact, the diagnostic tools we have, and the available therapeutic options.

## Epidemiology

Delirium is an acute and fluctuating impairment of attention, cognition, and behavior (Table 1). (5) Each of these elements is key to the proper diagnosis. *Acute*: delirium is a new and distinct event from any previous neuro-psychiatric pathology, such as dementia, neuroses, etc. *Fluctuating*: delirious patients may experience breakthroughs of lucidity, they are mystified at what they realize happened to them, and they fear its recurrence. Repeated episodes of delirium may have similar cognitive characteristics, unique to each patient. We do not have a definition of how long these lucid intervals have to be, to be no longer considered part of a single episode of delirium. *Attention*: easy distractibility and difficulty in focusing on a simple task are common prodromes of the upcoming delirium. Although these signs may be missed by the inexperienced or rushed clinicians, family members will often bring them to our attention. *Cognition*: impaired cognitive function can manifest both as the inability to recognize familiar persons or topics of conversation and as the onset of hallucinations and delusions that may become particularly elaborate as delirium progresses. *Behavioral* alterations vary widely, from the classical psychomotor agitation that may cause harm, to a deceivingly calm state of fear, to behaviors unusual for each patient that may lead family members to assert that ‘they cannot recognize their relative’. Delirium is prevalent among critically ill patients, particularly those that are intubated and mechanically ventilated. In medical, surgical, and trauma ICUs, patients who undergo mechanical ventilation have a prevalence of delirium as high as 80%. (6) The need for mechanical ventilation is likely a surrogate of severity of illness, but the tracheal intubation *per se*, as well as the tight fitting of a non-invasive ventilation mask, may produce sufficient distress (inability to talk, breathlessness, etc.) to trigger delirium. It is important to note that most mechanically ventilated ICU patients are not hypoxemic and may have not been hypoxemic at the time of intubation; the implication that hypoxemia is a common trigger of ICU or perioperative delirium is not based on robust clinical evidence. (7).

### Etiology

The cause of delirium often escapes the clinician, and it is likely to be multifactorial. In patients who exhibit multiple known predisposing factors (see below) delirium may develop as a result of one modifiable trigger, such as the absence of windows and daylight in an ICU cubicle. Conversely, the occurrence of multiple potential triggers at the same time, such as the stresses of major surgery, general anesthesia, and the administration of opiates, may elicit delirium in a patient with no known predisposing factors.

The biological bases of delirium are still largely unknown, but functional neuroimaging and other neuro-behavioral techniques are providing novel and often visual information on the activity of areas of the central nervous system involved in different types and phases of acute confusional states. (8) A number of theories of the pathogenesis of delirium focus on neurotransmission, inflammatory response, and the role of acute injuries on a predisposed background. (9). In this article, we will limit our review to the factors that are known to predispose to delirium and those that can be considered modifiable precipitating factors or ‘triggers’. These include (Table 2):

*Older age*. Patients above 65 years of age have a high incidence of delirium at all levels of hospitalization: 10 - 15% in the general ward, 15 - 50% postoperatively, and nearly 90% in the ICU. (1) What makes the elderly more susceptible to delirium may include cardiovascular and metabolic comorbidity, the intake of large numbers of prescribed medications, a higher sensitivity to acute stress, surgery, and anesthetics, and the prevalence of dementia and other chronic neurologic diseases.

*Dementia*. There seems to be a complex interaction between delirium and dementia. As many as two-thirds of elderly patients developing delirium have baseline dementia. (10,11) The progression of dementia seems to be accelerated by ICU delirium and postoperative delirium (also called ‘postoperative cognitive dysfunction’). (12)

*Severity if illness.* Delirium is not only more frequent in hospitalized patients with multiple medical problems, but it is often the early warning of the onset of a new complication, such as sepsis or congestive heart failure. A number of studies have confirmed the statistical association of indices of severity of illness such as the Acute Physiology And Chronic Health Evaluation Score II (APACHE II) with the development of delirium. (13,14)

*Hip Fractures*. Elderly individuals undergoing surgical fixation of a hip fracture have a particularly high incidence of perioperative delirium, possibly related to their advanced age and organic disorders, but also to anemia, pain, surgery and anesthesia, and to analgesic medications. (12,15) In this patient population, postoperative delirium is associated with a higher rate of dementia and mortality at 5 years.

Acute, modifiable risk factors include:

*Hypoxemia and cerebral hypoperfusion.* It seems logical maintaining hemodynamic and metabolic stability throughout a stressful period, be it major surgery or a critical illness to support brain metabolism and function. However, except probably for extreme event such as a cardiac arrest, the etiologic role of hemodynamic instability as it may occur during complex surgery is not so clear. A few studies have supported this relationship (15), others have not. (7)
*Benzodiazepines* have been consistently associated with the onset of delirium, with no exception among individual drugs, and possibly in a dose-dependent fashion. (6,14,16) However, they can be effective in special conditions. They are the drug of choice in benzodiazepine- and alcohol-withdrawal syndromes. In addition, they may be very valuable in severe hyperactive delirium, not to treat delirium *per se*, but to temporarily calm the patient and allow the implementation of other therapeutic measures.
*Opiates* also have been consistently associated with delirium. (14,16) Despite anecdotal reports of the contrary, there is no clear evidence of a μ-receptor agonist being different from another in triggering the onset of delirium. Just like for benzodiazepines, whose anxiolytic and sedative effect may be beneficial for delirious patients, the analgesic and sedative effect of opiates also may be immediately beneficial. However, these temporary advantages must be carefully balanced with the long-term effect on the patient mental status.

## Impact of delirium

Delirium in the ICU has been consistently associated with an increased duration of mechanical ventilation, increased ICU and hospital stay, and a higher mortality rate. (2,12,14) Recent findings also associate delirium to prolonged cognitive decline. (4) The obvious question is whether delirium *causes* these poor outcomes or it *occurs* in ICU patients that are particularly ill (see correlation with high APACHE II scores) and will naturally have a high rate of complications and death. Several lines of thought support a causal role of delirium in the ultimate demise of critically ill patients. *First*, delirious patients occasionally hurt themselves, through self-extubation, falls, etc. However, it is unclear whether such episodic events are sufficient to aggravate the overall prognosis of delirium. *Second*, delirious patients are exposed to the side effects of additional pharmacologic therapy, prolonged time on the ventilator, and may not participate in mobilization and physical therapy, leading inevitably to more nosocomial complications and, possibly, increased mortality. *Finally*, delirium could be a sign and a cause of ‘brain failure’ or ‘acute brain dysfunction’ (6), which, like any other organ failure, will increase the overall odds against recovery and survival. (3) However, as appealing as these views can be, they have only been supported by multivariate logistic regression analysis. The definite demonstration that delirium kills patients necessitates a biological mechanism, and the evidence that proper diagnosis and treatment of delirium decrease the mortality of critically ill patients. (17)

## Diagnosis

Diagnosing delirium is simple when the patient is agitated, delusional, and tirelessly resistant to reassurance and redirection. This is the classical ‘hyperactive delirium’, but not the only face of delirium, and possibly not the most common. Delirium is often subtle at the onset, when the patient becomes unable to focus on simple tasks such as memorizing a sequence of letters. However, he / she may already suffer of delusions that will reveal if skillfully probed. That’s when our patient discloses in a whisper that a certain nurse ‘has been trying to kill him every night’ or that she must go home now ‘to attend to serious business matters’. In certain patients, delirium remains quiet and devoid of any agitation. This ‘hypoactive delirium’ may be less frequently diagnosed because of its subtle manifestations, but is not less common; it seems more frequent in the elderly, and it has been repeatedly linked to a worse prognosis. (3,4,18) Most commonly, the same patient may alternate hyper- and hypoactive episodes (‘mixed’ delirium). The implication of a worse prognosis associated with one or the other subtype of delirium has to imply a different physiologic makeup, unless one hypothesizes that the hypoactive delirium is less frequently diagnosed and therefore less frequently treated, thus leading to an increased mortality. However, this line of reasoning implies that: *a)* we have an effective treatment for ICU delirium, and *b)* delirium *per se* kills patients, but for neither point we have robust evidence. Furthermore, the implication of a worse prognosis attached to hypoactive delirium has prompted vigorous calls for active seeking out delirium using systematically a validated diagnostic instrument as part of the routine nursing evaluation of all ICU patients. (3,18,19) This practice may or may not be the best-fitting solution for all ICUs, as we suggest it could be prevented with a thorough education of the ICU staff (see below).

It is clear that delirium is at least a complex and multifaceted syndrome that requires knowledge and experience to diagnose it. To complicate things, the most severely ill ICU patients, i.e., those who primarily develop delirium, can be averbal due to airway intubation. For decades, the inability to communicate by voice impeded the availability of a reliable and valid diagnostic instrument of delirium in the ICU, hindering our understanding and treatment of these patients. This came to an end with the development and validation of the Confusion Assessment Method for the ICU (CAM-ICU), a version of the already existing CAM applied to non-verbal ICU patients. (20) Other diagnostic instruments for ICU delirium have been developed, but the only one that has reached widespread use is the Intensive Care Delirium Screening Checklist (ICDSC). (21)

The CAM-ICU and the ICDSC are two similar scales, both developed on the diagnostic principles of DSM-IV (ref.) and both extensively validated. The CAM-ICU is easy to administer, and its 4 steps are tied to each other, so that it is common to be done after step 1 or 2. ^1^ The ICDSC contains more diagnostic criteria and may be suited for the analysis of different manifestations of delirium (22)

The ICDSC may also be less sensitive than the CAM-ICU. (23). A number of benefits have resulted from the availability of validated diagnostic instruments for delirium.

1. *Enhanced sensitivity.* We have a test that is much more sensitive than the empiric diagnosis of delirium available before. For example, al patients with dementia or any pre-existed neuro-psychiatric condition (e.g., bipolar disorder) use to be excluded from studies of ICU delirium. Since then, we have learned a great deal about the complex interaction between dementia and delirium, where the former may be a risk-factor for the latter, and the latter may accelerate the evolution of the former thus leading to an earlier severe dementia and need for institutionalization (see above, under ‘Etiology’).
2. *Higher specificity*. Not all emotional / psychological manifestations of critical illness are delirium. ICU patients are often in pain, anxious, ‘disoriented’ in an unfamiliar and intimidating environment, weakened and frustrated by the lack of sleep, the noise, and the overall loss of control of an unfamiliar experience. The administration of potent analgesic and sedative medications may temporarily affect memory and orientation, such that a patient may not always pass the time-honored ‘alert and oriented x 3’ test, or classic questions such as ‘who is the President of the United States?’. Yet, one wonders about the relevance of such questions if applied to those us who are experiencing a frustrating age-related selective decrease in memory of, e.g., names and places; or, when we consider what percentage of the population knows who their President is. ^2^
3. *The standardization of the diagnosis of delirium* that can be easily used in different ICUs, under different circumstances, and even in different languages throughout the world.
4. *The opportunity of using delirium as a quality indicator*. Delirium is common, frequently iatrogenic, and intimately linked to the process of care. As such, it is well-suited for use as a quality indicator. (24) Accordingly, it has already been included in lists of markers of quality and patient safety, such as those of the National Quality Measures Clearinghouse of the Agency for Healthcare Research and Quality.^3^

## Treatment

Treatment of delirium is still mainly symptomatic, as its underlying mechanism is often unidentified. A thorough search for a modifiable cause should always be performed: hypoxemia, serum sodium imbalance, uremia, hepatic failure, intoxications, etc. Even when a predominant factor is not identified, which is common, a number of generic contributing factors can often be corrected (see under ‘Etiology’).

*Environmental and behavioral interventions.* Predisposing factors specific to each patient include dementia, prior stroke or transient ischemic attack, depression, severe illness or multisensory impairment. Precipitating factors are related to external stimuli or insults such as major surgery, ICU stay, and sleep deprivation, multiple psychoactive or analgesic medications (Table 2).

A number of studies of delirium in hospitalized, non-ICU patients, has shown the effect of interventions aimed at preventing delirium by addressing known stressors, (25) summoning expert help (26) and implementing educational programs. (27) Recently, a similar study in concept tested the effectiveness of teaching and implementing interventions such as focused pain and analgesia management on a large cohort of ICU patients, resulting in superior patient comfort, shorter duration of mechanical ventilation, and a lower incidence of subsyndromal (i.e., mild) delirium. (28)

*Pharmacologic treatment*. Prophylaxis rather than treatment would be ideal for a syndrome that ‘feeds on its own progress’ to become more resistant to interventions, given the patients’ lack of cooperation and the frequent side effects of treatment. Although some data exist on the (partial) effectiveness of administering haloperidol or risperidone to patients at risk for the development of delirium (postoperative in these studies) no large controlled studies have been done in the ICU. (29,30)

First line of treatment of ICU delirium remains intravenous *haloperidol*, a butyrophenone antipsychotic recommended by guidelines of the Society of Critical Care Medicine, (19) and supported in surveys of intensivists and pharmacist. (31,32) Yet, haloperidol is not approved for intravenous use by the Food and Drugs Administration, has not been shown in a properly controlled study to decrease the morbidity of delirium, and is marred with significant side-effects, particularly ventricular arrhythmia, characteristically *Torsades de-points*. Close monitoring of the QT-interval during haloperidol is strictly recommended, although a precise safe range of duration of the QT-interval (corrected by the heart rate) has not been defined. Most clinicians would like to discontinue the drug with a QT-correct above 400 msec., but other factors such as underlying arrhythmia, the baseline duration of the QT (often close to 400 msecs. off haloperidol), and the presence of additional risk factors (quinolone antibiotics, amiodarone, hypomagnesemia) must all be taken in account. (33) Extrapiramidal motor symptoms can also occur, but rarely in the acute, intravenous phase of therapy.

Atypical antipsychotics such as *olanzapine* and *quetiapine* are also used both as prevention and as treatment of ICU delirium. These drugs have similar effects to haloperidol, but are not available for intravenous use. Similarly to haloperidol, they have not been tested on meaningful outcomes in large controlled trials, although some indication of their efficacy has been proven (34). Atypical anti-psychotics are not devoid of severe side-effects. Initially marketed as less prone to cause QT-interval abnormalities (35), this has still to be demonstrated properly. In non-ICU patients, the onset of malignant ventricular arrhythmia and sudden death was at least the same with atypical as with traditional antipsychotics such as butyrophenones and phenothiazines. (36)

*Benzodiazepines* (see under Etiology) are not recommended drugs to treat delirium, except as a temporary intervention to treat the severe psychomotor agitation of some patients. Conversely, benzodiazepines remain the drugs of choice in alcohol-withdrawal delirium.

*Dexmedetomidine*, a selective α-2-adrenergic agonist, is a more recent option for delirium management. Dexmedetomidine has the favorable profile of inducing mild sedation devoid of respiratory depression and seemingly producing a ‘mind-clearing effect’ that is not seen in the traditional gamma-amino-butyrate-mediated sedatives like benzodiazepines and propofol. Contrarily to the antipsychotocs, dexmedetomidine has been studied in large randomized trials and has compared favorably versus lorazepam and midazolam by decreasing the onset and duration of delirium (37,6). However, the effects of a widespread use of dexmedetomidine as prophylaxis / treatment of delirium are unknown. Anecdotally, the response to the sedative and ‘mind-clearing’ effects of dexmedetomidine is erratic, frequently limited by its predictable side effects of hypotension and bradycardia.

### Conclusions

Delirium is prevalent in the ICU. It prolongs the stay of mechanically ventilated patients, increases healthcare costs, and its neuropsychological sequelae may last for months- to years. We know a number of predisposing factors but we do not do a good job in eliminating them. Treatment is still rudimental, with drugs that have significant toxicity or limited efficacy, and we still resolve to using restrains. Awareness of ICU providers to the clinical significance of delirium has improved. Education rather than enforcement of untested practices (e.g., routine administration of a diagnostic scale) seem the logical approach.

## Figures and Tables

**Figure 1:**
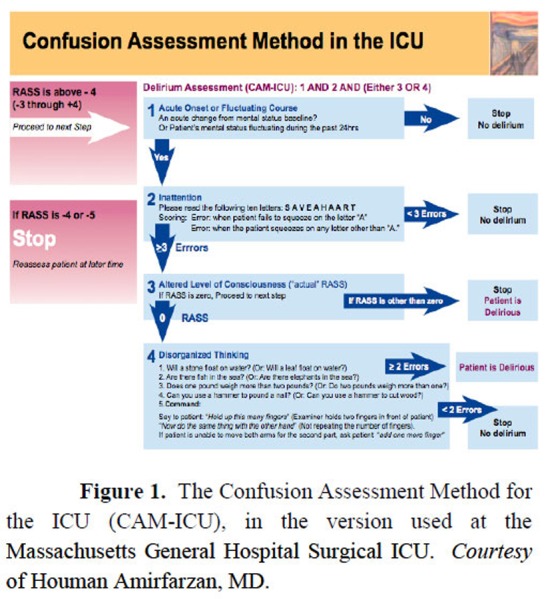
The Confusion Assessment Method for the ICU (CAM-ICU), in the version used at the Massachusetts General Hospital Surgical ICU. *Courtesy* of Houman Amirfarzan, MD.

**Table 1:** Diagnostic elements of delirium.

A disturbance of	
**Attention**	Difficulty focusing, easy distractibility
**Cognition**	Disorganized thinking; incoherent speech; hallucinations; delusions
**Behavior**	Agitation vs. lethargy; fear; paranoia; irritability
That is	
**Acute**	Occurs over a few hours, sometimes with short-lived prodromes
**Fluctuating**	Lucid intervals between episodes, with memory of the events

**Table 2:** Factors involved in the development of delirium.

**Predisposing factors**	
Older age	> 65 years, and continues to increase beyond it
Cognitive factors	Dementia, chronic psychosis, stroke
Functional status	Bedridden, debilitating stroke, dependency
General health status	Visual and hearing impairment, malnutrition
Chemicals	Psychoactive drugs Multiple drugs
Comorbidity	Cardiac disease, hepatic failure, uremia, multiple conditions
**Precipitating factors**	
Cerebral hypoperfusion	Severe hypotension, ‘low flow’ states, shock, hypoxemia
Acute stressors	Sepsis, surgery, anesthesia, major trauma, high fever
Withdrawal	Alcohol, benzodiazepines, opiates, other drugs of abuse
Benzodiazepines	All drugs of this class. Dose-dependent
Opiates	All agonists, and agonist-antagonist, though to a variable extent

**Table 3.** Elements of the Intensive Care Delirium Screening Checklist (ICDSC)

	**Day 1**	**Day 2**	**Day 3**
Altered level of consciousness *			
Inattention			
Disorientation			
Hallucinations, psychosis			
Psychomotor agitation or retardation			
		
Inappropriate speech, mood			
Altered sleep / wake cycle			

A. No response

B. Response only to intense and repeated stimuli

C. Response to mild / moderate stimuli

D. Normal wakefulness

E. Exaggerated response to normal stimuli

NOTE: If A or B, the evaluation is not completed.

## Footnotes

1 http://www.youtube.com/watch?v=6WyJ0zL7VkI&feature=g-hist&context=G1f4c9AHTs_GGQAAAA http://www.youtube.com/watch?v=fyaQuYnnoZ4&context=G1f4c9AHTs_GGQAAAA Both sites were accessed on December 4^th^, 2011.

2 http://www.people-press.org/2007/04/15/public-knowledge-of-current-affairs-little-changed-by-news-and-information-revolutions Last accessed on December 4^th^, 2011.

3 http://www.qualitymeasures.ahrq.gov last accessed on December 4^th^, 2011
